# Hierarchical Lotus-Seedpod-Derived Porous Activated Carbon Encapsulated with NiCo_2_S_4_ for a High-Performance All-Solid-State Asymmetric Supercapacitor

**DOI:** 10.3390/molecules28135020

**Published:** 2023-06-27

**Authors:** Siyi Cheng, Xiaowu Wang, Kang Du, Yu Mao, Yufei Han, Longxiao Li, Xingyue Liu, Guojun Wen

**Affiliations:** School of Mechanical Engineering and Electronic Information, China University of Geosciences, Wuhan 430074, China; chengsiyi@cug.edu.cn (S.C.);

**Keywords:** all-solid-state asymmetric supercapacitor, lotus seedpod, porous activated carbon, hierarchical structure, NiCo_2_S_4_

## Abstract

Converting biowaste into carbon-based supercapacitor materials provides a new solution for high-performance and environmentally friendly energy storage applications. Herein, the hierarchical PAC/NiCo_2_S_4_ composite structure was fabricated through the combination of activation and sulfuration treatments. The PAC/NiCo_2_S_4_ electrode garnered advantages from its hierarchical structure and hollow architecture, resulting in a notable specific capacitance (1217.2 F g^−1^ at 1.25 A g^−1^) and superior cycling stability. Moreover, a novel all-solid-state asymmetric supercapacitor (ASC) was successfully constructed, utilizing PAC/NiCo_2_S_4_ as the cathode and PAC as the anode. The resultant device exhibited exceptionally high energy (49.7 Wh kg^−1^) and power density (4785.5 W kg^−1^), indicating the potential of this biomass-derived, hierarchical PAC/NiCo_2_S_4_ composite structure for employment in high-performance supercapacitors.

## 1. Introduction

In recent decades, supercapacitors have been widely used in the start-up system of electric vehicles, electric buses, smart grids, portable electronic devices, and other systems [1,2,3]. However, supercapacitors still suffer from relatively lower energy density than lithium-ion batteries [4,5]. To improve the energy density of supercapacitors, one promising strategy is to develop a hybrid supercapacitor with a faradic and nonfaradic capacitive electrode, which could deliver a wide operational potential window to enhance the energy density [6]. Another effective approach is to fabricate a high-performance supercapacitor material structure with high surface area, high conductivity, superior structural stability, and porosity brought by the scale effects [7,8].

Carbon materials hold immense potential for supercapacitor applications owing to characteristics such as excellent conductivity, being easy to prepare, low cost, corrosion resistance, and good biocompatibility [9,10]. Various types of carbon materials, such as porous carbon [11,12], graphene [13], activated carbon (AC) [14,15], carbon nanotubes (CNTs) [16], and carbon nanofibers [17], have been extensively researched as supercapacitor materials. The preparation of the porous carbon structure could effectively improve the ion adsorption capability and shorten the ion transportation path, thus significantly improving the energy storage characteristics of the supercapacitor [18,19,20]. Among the various carbon materials, AC, with its high specific surface area, low price, and abundance, has been widely studied as a supercapacitor material [21]. The precursor material, heat treatment, and activation process are crucial for achieving a porous structure with high surface area [22]. Natural biomass materials, such as wood, grapefruit peel, coconut shell, agricultural waste, etc., are commonly employed in the preparation of porous carbon materials [23,24]. Lotus seeds, a popular leisure food in China, produce plenty of lotus seedpods as typical biological waste. Transforming this kind of biowaste into a high-performance energy storage material is of significance towards energy conservation, emission reduction, and environmental governance.

Nonetheless, the limited specific capacitance of carbon materials, which is attributed to their electrochemical double-layer capacity (EDLC) property, constrains their supercapacitor application [25]. Pseudocapacitance materials have higher energy density and better charge transfer characteristics than carbon materials due to their redox reactions [26]. Of different pseudocapacitive materials, NiCo_2_S_4_ has garnered significant attention due to its exceptional properties from the dual redox elements of Ni and Co [27,28]. However, relatively poor electrical conductivity and inadequate contact with electrolytes still limit its performance [29,30]. Combining NiCo_2_S_4_ with porous carbon to form a hybrid heterostructure could take advantage of both materials and provide better supercapacitor performance [30,31,32]. However, developing a high-performance AC/NiCo_2_S_4_ composite structure from biowaste which can fully utilize the advantages of these two kinds of material is still challenging.

**Figure 1:**
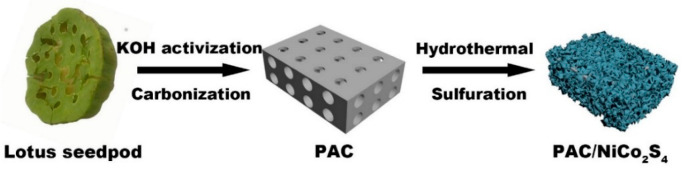
Schematic diagram of the preparation process of the PAC/NiCo_2_S_4_ composite structure.

In this work, a porous activated carbon (PAC) was fabricated with lotus seedpod as the raw material and KOH as the activator. The high-temperature carbonization–activation two-step method was employed to prepare lotus-seedpod-based PAC, which possesses a large specific surface area (Figure 1). Upon encapsulating the NiCo_2_S_4_, the PAC/NiCo_2_S_4_ composite structure demonstrated excellent pseudocapacitance characteristics, including remarkably high specific capacitance and long cycle life. An ASC was fabricated with PAC/NiCo_2_S_4_ as the electrode material, exhibiting superior behaviors in terms of high energy density, high power density, and good cycle stability.

## 2. Results and Discussion

### 2.1. Structure and Morphology Characterizations of the PAC/NiCo_2_S_4_ Composite

**Figure 2:**
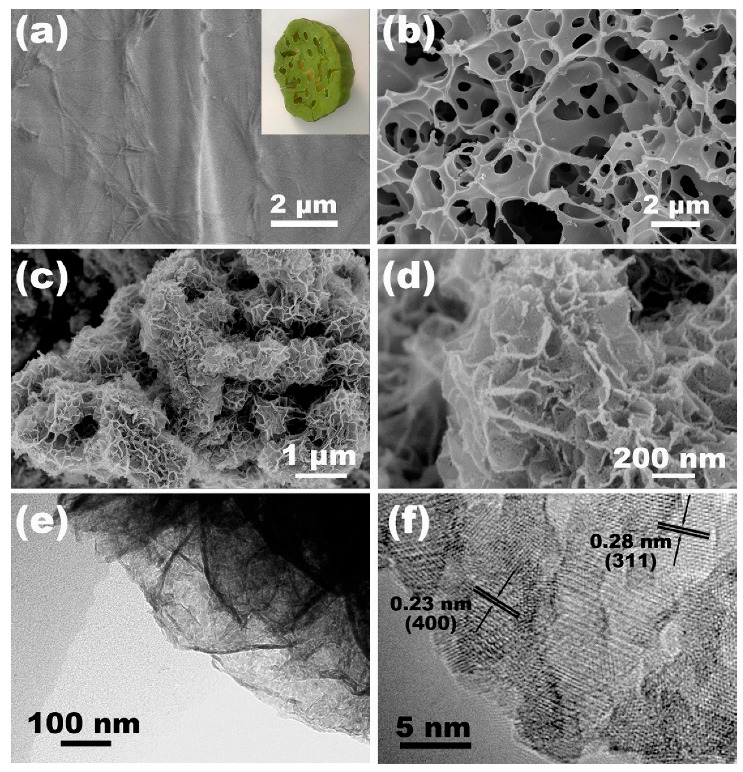
Low-magnification SEM images of the untreated lotus seedpod (**a**), the PAC (**b**), and the PAC/NiCo_2_S_4_ (**c**). (**d**) High-magnification SEM image of the PAC/NiCo_2_S_4_, (**e**) TEM, and (**f**) HRTEM images of the PAC/NiCo_2_S_4_. The inset of (**a**) is the photographic image of a lotus seedpod.

The inset picture in Figure 2a shows a photo of a lotus seedpod, which is a typical biowaste. Before the carbonization and activation treatment, the surface of the dried lotus seedpod shell was smooth; no obvious hollow structure could be found (Figure 2a). During the activation process, the molten KOH could easily penetrate the precarbonized material to create rich microporous and mesoporous structures. In addition, the higher content of oxygen-rich functional groups made it easier for the lotus seedpod precursor to make full contact with KOH to obtain a better activation effect [33]. This process can be explained as follows: KOH reacts with the pretreated lotus seedpod at a high temperature to generate H_2_O, CO, CO_2_, K_2_O, KCO_3_, and K ions, and then abundant holes are generated in carbon materials due to the gasification effect. In addition, K ions react with the carbon lattice to generate cross-linked potassium compounds, which are removed during subsequent cleaning and thus form amorphous porous carbon structures [34]. After the treatments, a mass of pores were found uniformly on the internal and external surfaces of the PAC (Figure 2b). A TEM image of the PAC further exhibits that the activated porous carbon material contains highly cross-linked pores (Figure S1a) with polycrystalline structure (Figure S1b). The combination of micro/nano porous structures on active porous carbon could facilitate the entrance of an electrolyte and ion transport path when used as supercapacitor material, thus improving the supercapacitve properties. Benefiting from the porous structure, the PAC could be uniformly covered by the interconnected sheetlike NiCo precursor (Figure S2a,b). After the sulfuration process, the PAC/NiCo_2_S_4_ composite structure could be maintained (Figure 2c), and the surface of NiCo_2_S_4_ is rough with porous morphology and a thickness of about 20 nm (Figure 2d). The porous morphology of the NiCo_2_S_4_ could be ascribed to the unequal diffusion of Ni^2+^, Co^2+^, and S^2−^ ions during the sulfuration process, which was also found in our previous work [35]. The microstructure of the PAC/NiCo_2_S_4_ composite structure was further observed by TEM. As shown in Figure 2e, NiCo_2_S_4_ exhibits a sheet structure composed of nanoparticles, and the dark spots in the TEM image indicate that these nanoparticles are curled and overlapped with each other, which is consistent with the SEM results above. The lattice spacing of 0.23 nm and 0.28 nm is in good agreement with the (400) and (311) crystal planes, respectively, of NiCo_2_S_4_ (Figure 2f) [32]. The integration of PAC with the hierarchically porous NiCo_2_S_4_ could heighten the specific surface area, thereby enabling efficient transportation of both ions and electrons within the electrode of the supercapacitor.

**Figure 3:**
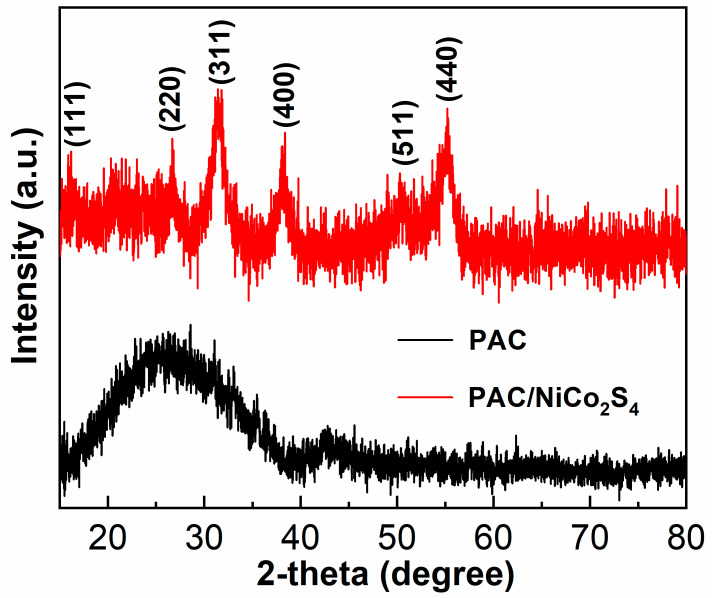
XRD patterns of the PAC and the PAC/NiCo_2_S_4_.

Figure 3 depicts the XRD spectra of the PAC and the PAC/NiCo_2_S_4_. The presence of graphitized carbon emanating from the PAC is demonstrated by the occurrence of broad peaks at 23.5° and 43.3°, corresponding to the (002) and (100) crystal planes, respectively [36,37]. These crystal phases indicate that the active porous carbon was mainly composed of amorphous disordered structures. Raman spectra further confirmed the disordered structure of the PAC. Figure S3 compares the Raman spectra of the KOH-activated PAC with that of unactivated carbon, where the preparation process of nonactivated porous carbon is similar to activated porous carbon except that it was not mixed with KOH before carbonization. Both carbon samples manifest two prominent peaks situated at 1346 and 1590 cm^−1^, which align with the D and G peaks of the carbon substance, respectively [38,39,40]. The I_D_/I_G_ intensity ratio, which corresponds to peak D to peak G, has been found to be associated with the degree of amorphousness and level of defects [41]. Notably, the intensity ratio between activated carbon and untreated carbon is 0.76 and 0.99, respectively, suggesting that after undergoing the activation process, the activated carbon exhibits a higher degree of defects. With regard to the XRD pattern of PAC/NiCo_2_S_4_, it is notable that all the identifiable characteristic peaks are in line with the indexed peaks of NiCo_2_S_4_ (JCPSD No. 23-1390). Specifically, the crystal phases at 16.5°, 27.2°, 31.9°, 38.6°, 50.8°, and 55.6° could be ascribed to (111), (220), (311), (400), (511), and (440), respectively. However, the characteristic peak of carbon is not obviously observed, which may be because the active porous carbon is completely wrapped by NiCo_2_S_4_, referring to the morphological characteristics shown in SEM images [42,43].

**Figure 4:**
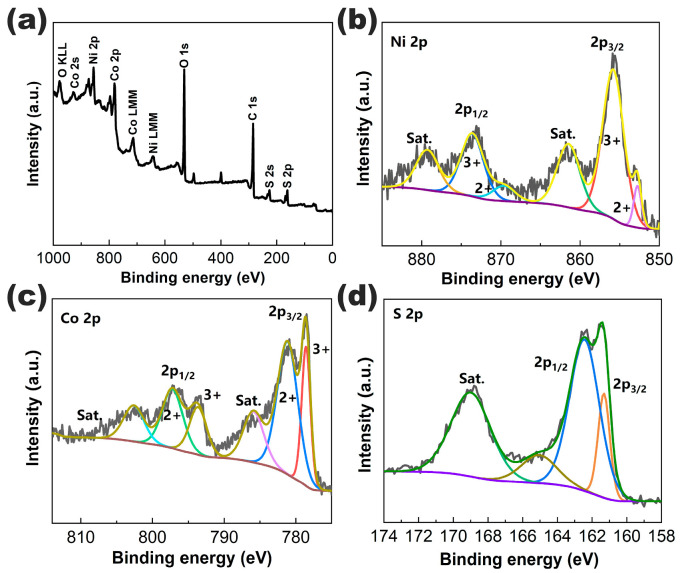
XPS spectra of the PAC/NiCo_2_S_4_: (**a**) survey spectrum, (**b**) Ni 2p, (**c**) Co 2p, and (**d**) S 2p. (For the Ni 2p and Co 2p spectra, 2+ and 3+ are referred to Ni^2+^/Co^2+^ and Ni^3+^/Co^3+^ oxidation states, respectively, while Sat. stands for shake-up satellite).

The elemental composition and valence state of the PAC/NiCo_2_S_4_ composite structure were studied by XPS. The XPS spectra of Ni, Co, and S have been fitted using the Shirley model. In the survey spectrum illustrated in Figure 4a, cobalt, nickel, sulfur, carbon, and oxygen elements could be found. Figure 4b exhibits the high-resolution Ni 2p spectrum, in which Ni 2p_3/2_ contains two spikes at 852.8 eV and 855.8 eV, which are attributed to Ni^2+^ and Ni^3+^, respectively [44]. The two peaks of Ni 2p_1/2_ at 869.6 eV and 873.6 eV also reveal the coexistence of Ni^2+^ and Ni^3+^. In addition, two strong satellite peaks in the spectrogram indicate that Ni^3+^ is the main component of the Ni element. As for the Co 2p spectrum in Figure 4c, two peaks located at 793.6 eV and 778.6 eV can be attributed to Co^3+^, while the other two peaks located at 781 eV and 797.2 eV can be ascribed to Co^2+^ ions. These results reveal that there are bivalent and trivalent Ni and Co elements in the NiCo_2_S_4_ compound, and Ni^3+^ and Co^2+^ are the main components. The element sensitivity factor of Ni 2p is 14.2 and that of Co 2p is 3.8, so the ratio of Ni 2p to Co 2p in the compound is 1.6:3.4, further proving that the compound is NiCo_2_S_4_. Figure 4d shows the high-resolution spectrum of S 2p. The S 2p spectrum can be divided into three main peaks and one shake-up satellite. The peak at 161.4 eV is characteristic of S^2−^. The component at 165 eV is typical of metal–sulfur bonds, while the component at 162.5 eV can be attributed to the sulfur ion in low coordination at the surface. The binding energy at 168.9 eV corresponds to the shake-up satellite [45]. According to the XPS analysis, the near-surface of the NiCo_2_S_4_ sample has a composition of Co^2+^, Co^3+^, Ni^2+^, Ni^3+^, and S^2−^, which is in good agreement with the NiCo_2_S_4_. Uniformly integrating the NiCo_2_S_4_ on porous carbon to form the PAC/NiCo_2_S_4_ composite structure represents a potential strategy for enhancing the cyclic properties of the material.

**Figure 5:**
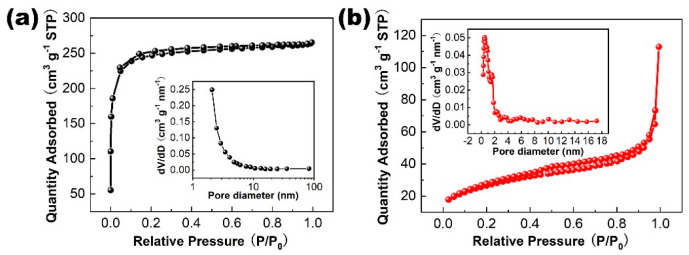
N_2_ adsorption–desorption isotherms of the PAC (**a**) and the PAC/NiCo_2_S_4_ (**b**) Insets: the corresponding pore-size distributions.

N_2_ adsorption–desorption isotherms and the corresponding pore-size distribution curve determined by a QSDFT equilibrium model is shown in Figure 5. The constant temperature adsorption and desorption curve of the PAC shows a type I isotherm (Figure 5a), in which the nitrogen adsorption capacity demonstrates a rapid increase at a low relative pressure, followed by a stabilization at a high relative pressure. [46]. In addition, the isotherms exhibit H4-type hysteresis loops at relative pressures of 0.08 and 0.99, which can be attributed to the mesoporous structures of the PAC. The pore-size distribution is also in line with mesoporous carbon (2–50 nm) [47]. The specific surface area of the PAC is 908.9 m^2^/g, with a pore volume of 0.41 cm^3^/g. The high specific surface area and mesoporous architecture of the material facilitate the entrance of electrolyte and ion transportation, thus improving the supercapacitive properties. After the integration of the NiCo_2_S_4_, the isotherm curve changes from a type I to a type II isotherm (Figure 5b), as well as the H4 type of hysteresis loop and pore-size distribution, suggesting that the material mainly consist of micropores [46,48]. Inside the hierarchical structure, ion transport through NiCo_2_S_4_ to carbon along with NiCo_2_S_4_ provide more electro-active sites than carbon; this way of combination is effective in improving redox reactions. The specific surface area of the PAC/NiCo_2_S_4_ composite could be calculated as 79.3 m^2^/g. The high surface area and hierarchical porous structure could potentially enhance the pathway for ion/electron conduction and augment the quantity of active sites, leading to an enhancement in the electrochemical property of the supercapacitor.

### 2.2. Electrochemical Measurements of the PAC/NiCo_2_S_4_ Electrode

**Figure 6:**
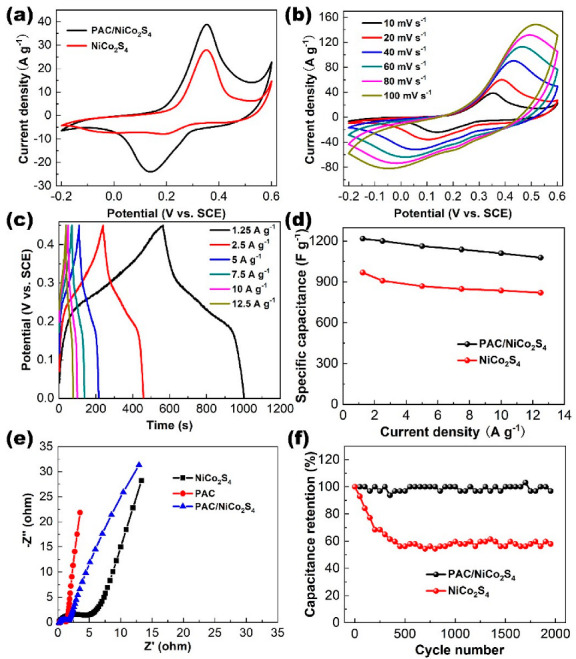
(**a**) Comparison of CV curves for the NiCo_2_S_4_ and the PAC/NiCo_2_S_4_ electrodes recorded at a scan rate of 10 mV s^−1^. (**b**) CV and (**c**) galvanostatic charge–discharge curves of the PAC/NiCo_2_S_4_ electrode at various scan rates and current densities. (**d**) Specific capacitance of the NiCo_2_S_4_ and the PAC/NiCo_2_S_4_ electrodes at different current densities. (**e**) EIS spectra of the PAC, the NiCo_2_S_4_, and the PAC/NiCo_2_S_4_ electrodes. (**f**) Cycling performances of the NiCo_2_S_4_ and the PAC/NiCo_2_S_4_ electrodes at the current densities of 5 A g^−1^.

In order to examine the electrochemical characteristics of the PAC/NiCo_2_S_4_ composite structure utilized as the electrode material for an ASC device, a three-electrode test was performed. Figure 6a reveals the CV (cyclic voltammetry) curves of the PAC/NiCo_2_S_4_ and the bare NiCo_2_S_4_ at 10 mV s^−1^. The PAC/NiCo_2_S_4_ depicts a pair of redox peaks at 0.38 V and 0.14 V, respectively, revealing that the pseudocapacitance characteristics associated with Co^2+^/Co^3+^/Co^4+^ and Ni^2+^/Ni^3+^ dominate the electrochemical properties of the electrode materials [49,50]. The PAC/NiCo_2_S_4_ electrode possesses a larger CV curve area than the pure NiCo_2_S_4_, proving that the introduction of the PAC as a load could improve the energy-storage performance. The CVs of the PAC/NiCo_2_S_4_ across a range of scan rates extending from 10 mV s^−1^ to 100 mV s^−1^ is shown in Figure 6b. The morphology of the CV curves remains largely unaltered, indicating electrochemical reversibility and favorable rate properties of the the PAC/NiCo_2_S_4_ electrode. Moreover, the CV curves of the PAC exhibit a rectangle-like profile at various scan rates (Figure S4), indicating an EDLC-type property.

The GCD results of the PAC/NiCo_2_S_4_ electrode at current densities ranging from 0 to 0.45 V are shown in Figure 6c. The nonlinear curve shape and the existence of the voltage plateau further prove the pseudocapacitive character of the PAC/NiCo_2_S_4_. The shape of the GCD curve remains almost unchanged with the increase in current density, revealing that the material has good reversible electrochemical properties. The specific capacitance of the PAC/NiCo_2_S_4_ are 1217.2, 1200.1, 1162.5, 1137.8, 1110, and 1078.1 F g^−1^ at current densities of 1.25, 2.5, 5, 7.5, 10, and 12.5 A g^−1^, respectively (Figure 6d), which are significantly higher than the comparable NiCo_2_S_4_ values. By incorporating the PAC, the conductivity of the composite structure was improved, which facilitated the ion/electron transportation pathways and resulted in an increase in capacitance. It is noteworthy that the specific capacity attenuates only 11% with an increased current density from 1.25 A g^−1^ to 12.5 A g^−1^, indicating an excellent rate capability of the material. The Nyquist plots of all three electrodes demonstrate the presence of a semicircular pattern at high frequencies and a linear trend at low frequencies. (Figure 6e). The smaller semicircle of the PAC/NiCo_2_S_4_ reveals a smaller charge transfer impedance, that is, the composite material possesses a great electrochemical active surface area [43]. Meanwhile, the slope of the PAC/NiCo_2_S_4_ curve is between the NiCo_2_S_4_ and the PAC, manifesting that the combination of the PAC and the NiCo_2_S_4_ could facilitate the ion diffusion resistance [51]. The cycling efficiency of the electrodes composed of PAC/NiCo_2_S_4_ and NiCo_2_S_4_ were investigated using a series of repeated GCD measurements at a specific current density of 5 A g^−1^. (Figure 6f). The capacitance of the composite electrode exhibited a gradual decrease, yet still maintained a retention rate of 96.8% after a total of 2000 cycles. This proved to be superior to the pure NiCo_2_S_4_ electrode. The enhanced electrochemical efficacy of the PAC/NiCo_2_S_4_ composite is likely attributable to its unique hierarchical nanostructure, which seamlessly integrates with a highly porous carbon structure.

### 2.3. Electrochemical Measurements of the PAC/NiCo_2_S_4_//PAC ASC

**Figure 7:**
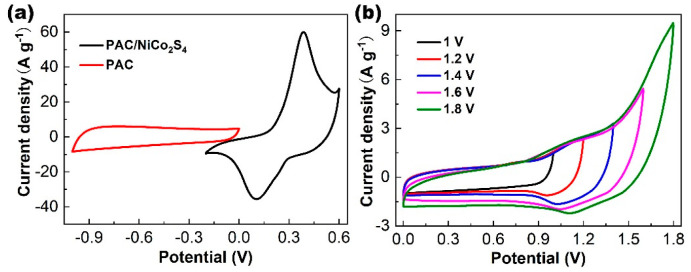
(**a**) CV curves of the PAC and the PAC/NiCo_2_S_4_ electrodes. (**b**) CV curves of the assembled PAC/NiCo_2_S_4_//PAC asymmetric supercapacitor tested at different potential windows.

An ASC was fabricated to assess the PAC/NiCo_2_S_4_ performance. In order to maximize the efficiency of asymmetric supercapacitors, it is imperative to achieve a balance in the mass of active materials present in both electrodes. Figure 7a shows the CVs of the PAC/NiCo_2_S_4_ and the PAC electrodes in the corresponding voltage range, which are 0 to 0.6 V and −1 to 0 V, respectively. The PAC electrode demonstrates a characteristic EDLC behavior, with a rectangular shape and the absence of discernible redox peaks. Meanwhile, the PAC/NiCo_2_S_4_ electrode exhibits distinct redox peaks reflecting its pseudocapacitive property. Hence, this suggests that these two electrode configurations can effectively function within a voltage range spanning from 0 to 1.6 V. Figure 7b illustrates the CV plots obtained from the PAC/NiCo_2_S_4_//PAC supercapacitor operating under various voltage windows from 0–1 V to 0–1.8 V. The area increases when the upper voltage increases from 1 V to 1.8 V, corresponding to the increase in the capacity of the supercapacitor. However, there is an obvious oxygen evolution reaction peak caused by hydrolysis when the voltage increases to 1.8 V, indicating that the working electrode will be unstable in this voltage range. Therefore, 0 to 1.6 V is selected as the optimal working voltage window of the PAC/NiCo_2_S_4_//PAC asymmetric supercapacitor.

**Figure 8:**
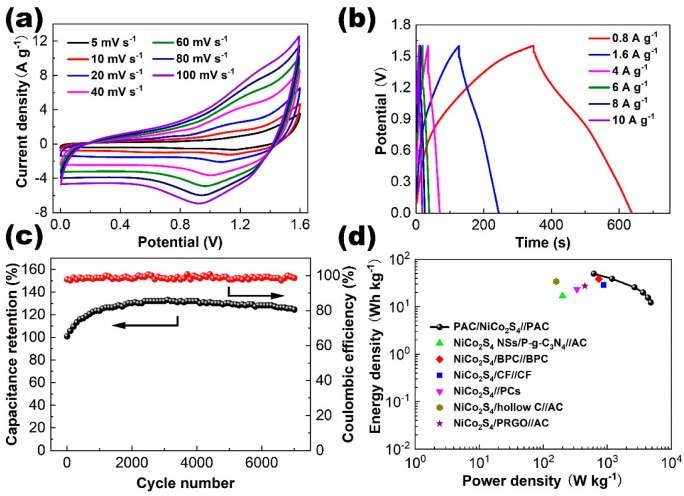
(**a**) CV and (**b**) GCD curves of the PAC/NiCo_2_S_4_//PAC ASC at various scan rates and current densities. (**c**) Cycling stability of the supercapacitor device at 4 A g^−1^. (The black and red lines are referred to stability and coulombic efficiency, respectively.) (**d**) Ragone plot of the supercapacitor device.

The CV curves of the PAC/NiCo_2_S_4_//PAC ASC working at various scan rates is shown in Figure 8a. The CV curves manifest quasi-rectangular shapes characterized by broad redox peaks, which indicate the concurrent contributions of EDLC and pseudocapacitance. The morphology of the CV profiles can be preserved when the scan rate is enhanced, indicating the superior rapid charge and discharge capability of the ASC device. Moreover, the GCD curves (Figure 8b) of the PAC/NiCo_2_S_4_//PAC ASC under different current densities show similar shapes with distinct voltage plateaus, also indicating good rate performance and pseudocapacitance characteristics. The specific capacitance could be computed to be 151.1, 126.7, 104.3, 99.6, 94.73, and 96.4 F g^−1^ at 0.8, 1.6, 4, 6, 8, and 10 A g^−1^, respectively, as shown in Figure S5. In addition, the Nyquist plot of the ASC device is depicted in Figure S6. The occurrence of a vertical line indicates the manifestation of Warburg resistance, attributed to the ionic diffusion inside the electrolyte. The suberect line observed suggests a distinctively capacitive response. The diminutive intercept located on the Z real axis shown in the high-frequency realm serves as an indicator of low equivalent series resistance.

The cycling stability of the PAC/NiCo_2_S_4_//PAC ASC was evaluated at 4 A g^−1^. During the charge–discharge process, the capacitance increases gradually in the initial 2000 cycles and remains stable in the following 5000 cycles. After 7000 cycles, the capacitance increases by 24.5% compared with the initial value (Figure 8c), which might be due to the electrode material being gradually infiltrated by solid electrolyte. Hence, the diffusion of electrolyte ions within the electrode material progressively enhances and creates more ion transport channels [52,53,54]. In addition, the porous structure promotes the activation of the material during the cycling, that is, the effective active area is increased and reaches its maximum at about 2000 cycles. The coulombic efficiency remains at about 100%, which further proves the highly reversible properties of the ASC device.

As shown in the the Ragone plot (Figure 8d), the as-prepared ASC exhibits a high energy density of 49.7 Wh kg^−1^ at the power density of 615.4 W kg^−1^, and retains a high power density of 12.3 Wh kg^−1^ even when the power density increases to 4785.5 W kg^−1^. The results of our study could be considered comparable or even superior in comparison to those reported in previous studies pertaining to NiCo_2_S_4_-based ASC devices, such as NiCo_2_S_4_ nanosheets/P-g-C_3_N_4_//AC (16.7 Wh kg^−1^ at 200 W kg^−1^) [55], NiCo_2_S_4_/biomass porous carbon//biomass porous carbon (38.5 W h kg^–1^ at 738.1 W kg^–1^) [56], NiCo_2_S_4_/carbon fiber//carbon fiber (28.8 Wh kg^−1^ at 878.3 W kg^−1^) [31], NiCo_2_S_4_//popcorn-derived activated carbon (23.3 Wh kg^−1^ at 335.8 W kg^−1^) [57], NiCo_2_S_4_/hollow carbon//AC (34.1 Wh kg^−1^ at 160 W kg^−1^) [58], and NiCo_2_S_4_/phytic acid modified reduced graphene oxide//AC (27.5 Wh kg^−1^ at 447 W kg^−1^) [59], highlighting the potential practical utilization of the PAC/NiCo_2_S_4_ composite material.

### 2.4. Discussion

The exemplary electrochemical properties of the PAC/NiCo_2_S_4_//PAC asymmetric supercapacitors could be attributed to the following factors: (1) Highly porous carbon binds to NiCo_2_S_4_ with microporous structures, providing a mass of active sites for pseudocapacitance redox reactions. (2) The active porous carbon has better conductivity than NiCo_2_S_4_, so the composite structure can take advantage of both the superior electrical conductivity of the PAC and the high electrochemical activity of NiCo_2_S_4_. This synergistic effect makes the composite structure have better electrochemical performance. (3) The PAC with good dispersion could avoid the aggregation effect of bare NiCo_2_S_4_, so the uniformly dispersed electrode materials can provide a good ion and electron transformation channel. (4) The PAC is uniformly coated with NiCo_2_S_4_ nanostructure, which enhances the mechanical and electrochemical stability of the composite structure. Therefore, the cycling stability of the electrode is significantly improved.

## 3. Materials and Methods

### 3.1. Synthesis of the Lotus-Seedpod-Based PAC

The lotus shell used in the experiment was collected from waste after eating lotus seeds. The lotus seedpod was dried at 60 °C for overnight, and the internal flocculent was then retained for subsequent activation and carbonization treatment. The specific treatment process was as follows: First, the crushed lotus seedpod shell was pretreated in Ar atomosphere at 350 °C for 2 h. Subsequently, the precarbonized sample was pulverized and blended with KOH at a mass ratio of 1:4. The amalgamated sample was then annealed at 800 °C in Ar atmosphere for 3 h. Following this, the activated carbon powder was thoroughly cleaned with HCl and DI (deionized) water to eliminate any residual KOH, before being dried for collection.

### 3.2. Synthesis of the PAC/NiCo_2_S_4_ Composite Structure

First, 0.145 g of nickel nitrate hexahydrate, 0.291 g of cobalt nitrate hexahydrate, 0.07 g of hexamethylenetetramine, and 0.015 g of trisodium citrate were dissolved evenly in 80 mL of DI water. Subsequently, 30 mg of PAC was added gradually to the solution and agitated vigorously for 1 h. The homogenously agitated amalgam was subsequently transferred into a 100 mL autoclave and subjected to a reaction at 100 °C for 6 h. After cooling, the PAC/Ni-Co precursor was cleaned and collected several times through centrifugation and ultrasolication. Then, 5 g of Na_2_S·9H_2_O was dissolved in 80 mL of DI water and stirred uniformly. The nickel–cobalt precursor/PAC prepared in the previous step was then slowly added to the Na_2_S solution, which was stirred for 0.5 h. The solution was subsequently subjected to reaction in a 100 mL autoclave at 160 °C for 8 h. Finally, the PAC/NiCo_2_S_4_ composite structure was obtained via repeated centrifugation and ultrasolication. The preparation process of the PAC/NiCo_2_S_4_ composite structure is illustrated in Figure 1. For comparison, NiCo_2_S_4_ was also synthesized in a similar process of PAC/NiCo_2_S_4_ without adding PAC during the hydrothermal process.

### 3.3. Material Characterizations

Field emission scanning electron microscopy (FESEM, FEI Nova NanoSEM 450, FEI, Hillsboro, OR, USA) and high-resolution transmission electron microscopy (HRTEM, FEI Tecnai G2 S-Twin, FEI, Hillsboro, OR, USA) were used to obtain the micro-morphologies of the materials. The crystal structure was characterized via powder X-ray diffraction (XRD, PW3040/60, Malvern Panalytical, Malvern, UK), with CuKα rays (~0.15406 nm) as the light source. X-ray photoelectron spectroscopy (XPS; PHI 5000 Versa Probe, ULVAC-PHI, Kanagawa, Japan) under Al Kα (1486.6 eV) irradiation at 25 W and 6.7 × 10^−8^ Pa was used to confirm the chemical composition and binding energy of the PAC/NiCo_2_S_4_, the wide scan measured as pass energy of 187.85 eV in the range of 10–1100 eV with a step size of 1.0 eV, and the narrow scan measured as pass energy of 58.70 eV for Ni 2p, Co 2p, and S 2p with a step size of 0.1 eV. A specific surface area and porosity tester (ASAP 2020, Micromeritics, Norcross, GA, USA) was utilized with N_2_ adsorption–desorption isotherms to analyze the porous property of the materials. The Brunauer–Emmett–Teller (BET) equation helped to calculate the specific surface area of the material.

### 3.4. Electrochemical Measurements

The positive electrode was fabricated by mixing PAC/NiCo_2_S_4_ composite structure, polyvinylidene fluoride (PVDF), and carbon black in DMF solvent at 8:1:1. Subsequently, the homogenized slurry was coated onto the nickel foam, which had been cleaned by HCl, followed by a vacuum-drying and -press treatment. The mass loading of the PAC/NiCo_2_S_4_ was estimated to be around 2 mg cm^−2^. A three-electrode configuration was established, with PAC/NiCo_2_S_4_, SCE, and Pt foil as the working, reference, and counter electrodes, respectively. An electrochemical workstation (PGSTAT-302N, Eco Echemie B.V. Company, Utrecht, The Netherlands) was used to conduct cyclic voltammetry (CV) and electrochemical impedance spectroscopy (EIS) measurements, whereby the EIS tests were conducted within the frequency range of 0.01 Hz to 100 kHz with alternating current (AC) amplitude of 10 mV. A charge–discharge tester (CT2001D, LAND Electronics Co., Ltd., Wuhan, China) was applied for the galvanostatic charge–discharge (GCD) tests. The specific capacitance of the PAC/NiCo_2_S_4_ composite structure was calculated using the equation:

(1) $$C_{s}=I∆t/m∆V$$

where $C_{s}$ represents the specific capacitance (F g^−1^), I stands for the discharge current (A), t is the discharge time (s), V is the discharge voltage (V), and m is the mass of the active material (g).

### 3.5. Preparation of All-Solid-State Asymmetric Supercapacitor

An ASC device was fabricated utilizing the PAC/NiCo_2_S_4_ and the PAC as the positive and negative electrodes, respectively. To optimize the performance, the charges in the two electrodes should be balanced:

(2) $$\frac{m_{+}}{m_{-}}=\frac{C_{S-}∆V_{-}}{C_{S+}∆V_{+}}$$

where m stands for the mass of active material (g), $C_{S}$ is the specific capacitance (F g^−1^), and *V* is the voltage range (V). The specific capacitance of the electrodes could be computed by GCD results.

To construct an ASC, the prepared electrodes and filter paper were immersed in PVA–KOH solution separately for 15 min, after which they were assembled into a sandwich structure. To evaluate the electrochemical performance of the ASC device, energy density (*E*, Wh kg^−1^) and power density (*P*, W kg^−1^) were computed though the following equations

(3) $$E={C\left(∆U\right)}^{2}/7.2$$

(4) P=3600E/∆t

## 4. Conclusions

In this work, the hierarchical PAC/NiCo_2_S_4_ composite structure was successfully fabricated through integrating NiCo_2_S_4_ on biomass-derived porous carbon. The PAC/NiCo_2_S_4_ material possesses a significant high specific capacitance (1217.2 F g^−1^ at 1.25 A g^−1^) and remarkable cycle stability, which can be attributed to its unique hierarchically compositional properties and hollow architecture. The PAC/NiCo_2_S_4_-based ASC device delivered a high energy density (49.7 Wh kg^−1^) and power density (4785.5 W kg^−1^). In addition, the ASC also showed excellent cycle stability and reversible electrochemical characteristics. Thus, the utilization of PAC produced from biomass as a conductive substrate for fostering pseudocapacitive materials displays considerable potential for the creation of superior asymmetric supercapacitors suitable for practical energy storage applications.

## Data Availability

The data presented in this study are available in the article and can be shared upon request.

## Supplementary Materials

The following supporting information can be downloaded at: https://www.mdpi.com/article/10.3390/molecules28135020/s1, Figure S1: (a) TEM and (b) HRTEM images of the PAC; Figure S2: (a) Low- and (b) high-magnification SEM images of the PAC/NiCo precursor; Figure S3: Raman spectra of the untreated carbon and the PAC; Figure S4: CV curves of the PAC at different scan rates; Figure S5: Specific capacitances of the PAC/NiCo_2_S_4_//PAC asymmetric supercapacitor at different current densities; Figure S6: Nyquist plot of the PAC/NiCo_2_S_4_//PAC asymmetric supercapacitor device; the inset is the enlarged Nyquist plot.

## Sample Availability

Samples of the compounds are available from the authors upon request.
