# Supplementary material for: Hierarchical Lotus-Seedpod-Derived Porous Activated Carbon Encapsulated with NiCo2S4 for a High-Performance All-Solid-State Asymmetric Supercapacitor
Source: Molecules. 2023 Jun 27;28(13):5020. doi: 10.3390/molecules28135020 (PMC10343735; doi:10.3390/molecules28135020)
Supplement: Supplementary file 1 [file molecules-28-05020-s001.zip › molecules-2391810-supplementary.pdf]

Supporting Information

Hierarchical Lotus Seedpod-Derived Porous Activated Carbon Encapsulated with $NiCo_2S_4$ for High-Performance All-Solid-State Asymmetric Supercapacitor

Siyi Cheng, Xiaowu Wang, Kang Du, Yu Mao, Yufei Han, Longxiao Li, Xingyue Liu and Guojun Wen*

[1] School of Mechanical Engineering and Electronic Information, China University of Geosciences, Wuhan 430074, China

* Correspondence: wenguojun@cug.edu.cn; Tel.: +86-27-67883421

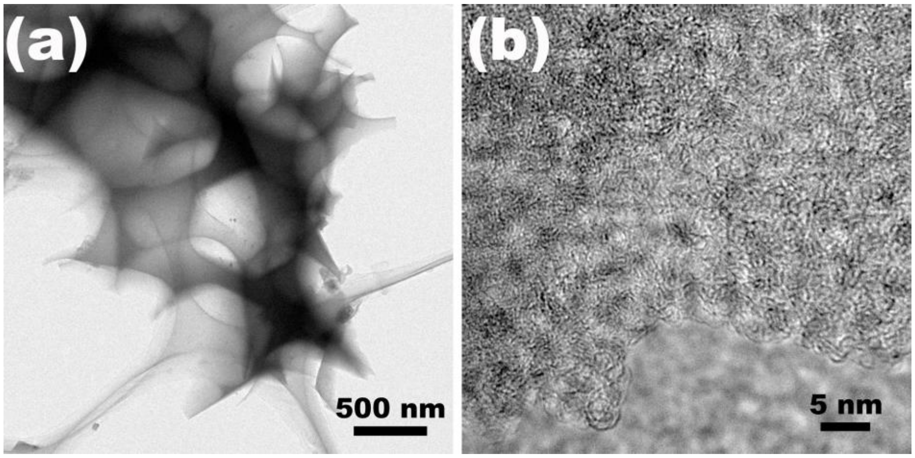

Figure S1. (a) TEM and (b) HRTEM images of the PAC.

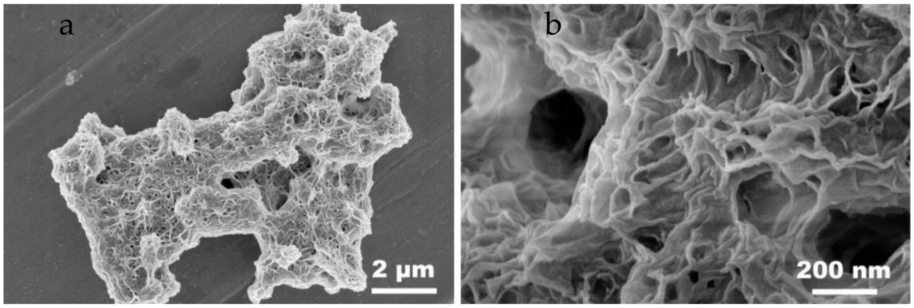

Figure S2. (a) Low and (b) high magnification SEM images of the PAC/NiCo precursor.

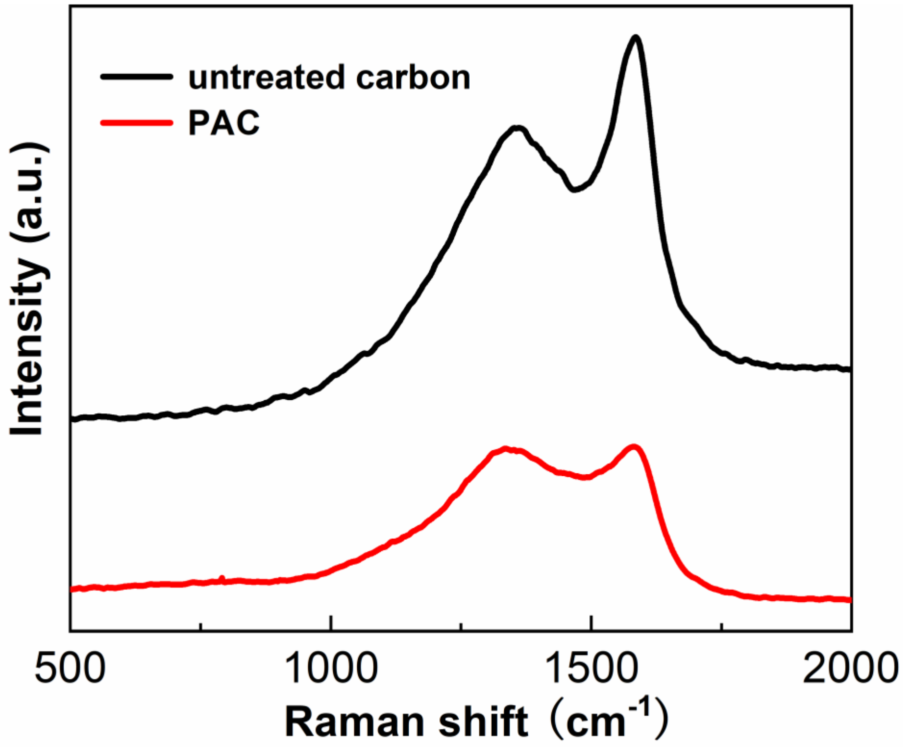

Figure S3. Raman spectra of the untreated carbon and the PAC.

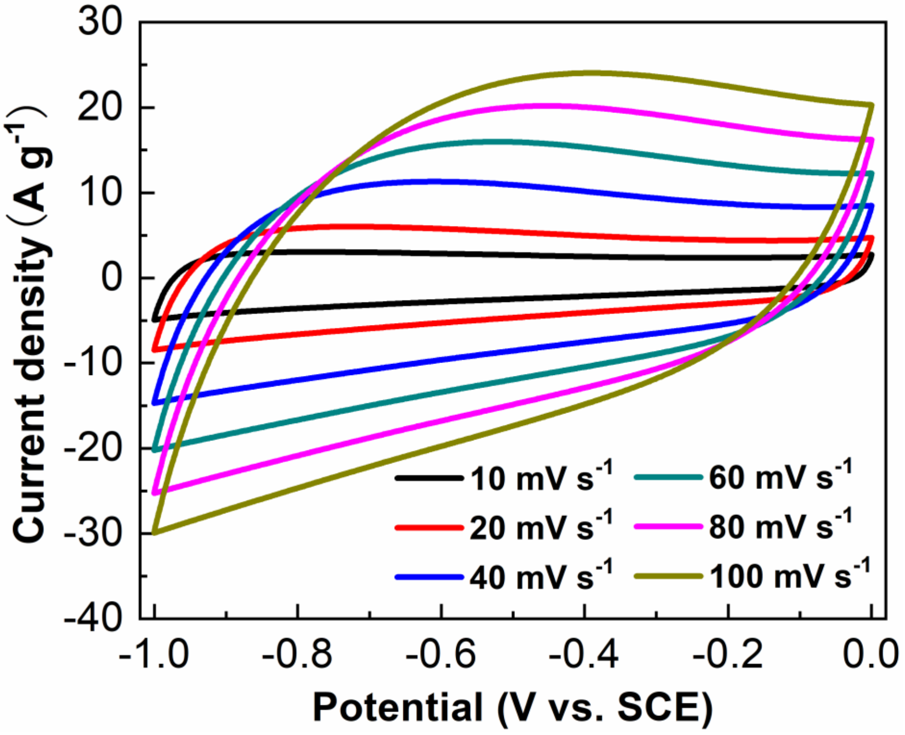

Figure S4. CV curves of the PAC at different scan rates.

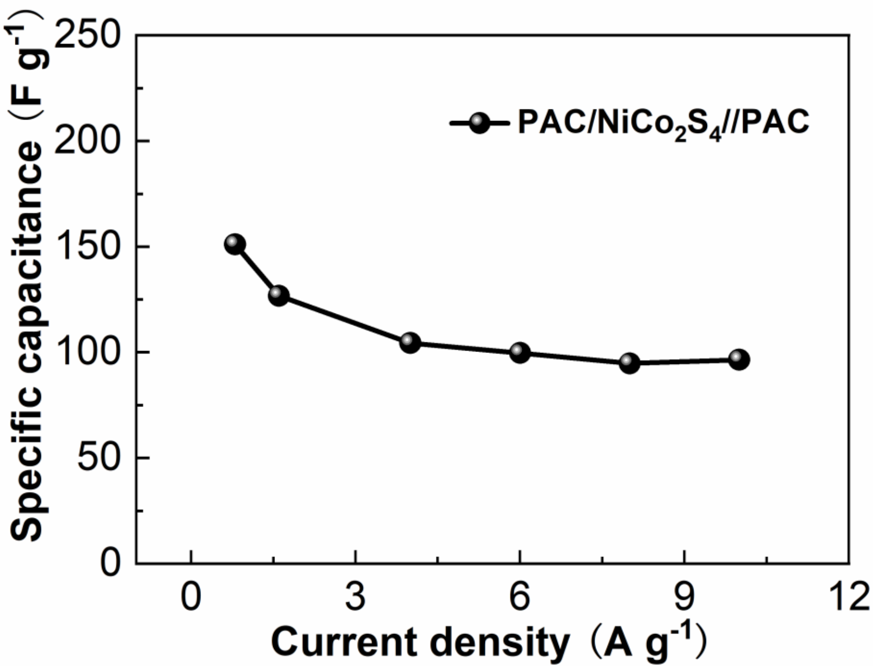

Figure S5. Specific capacitances of the PAC/$NiCo_2S_4$//PAC asymmetric supercapacitor at different current densities.

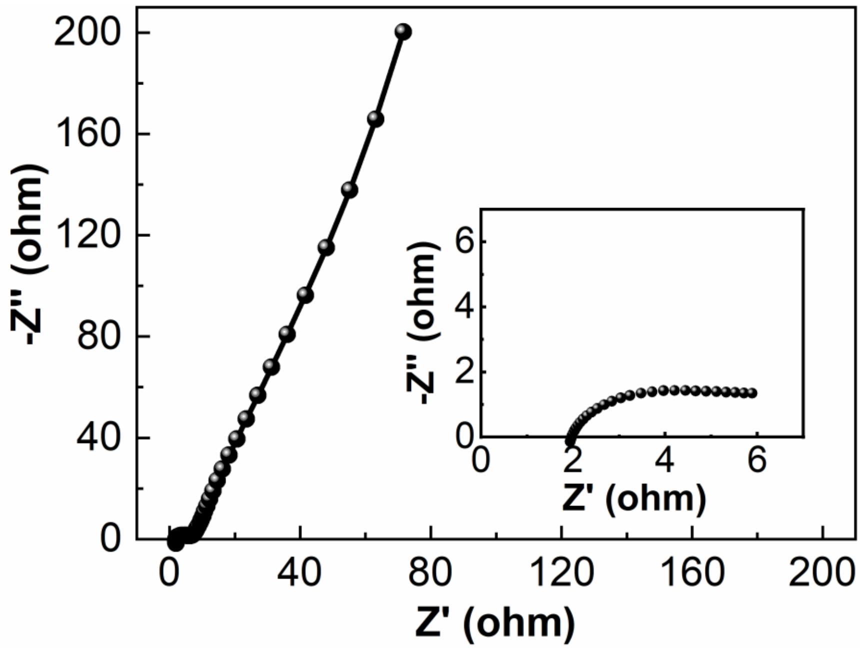

Figure S6 Nyquist plot of the PAC/$NiCo_2S_4$//PAC asymmetric supercapacitor device; the insets is the enlarged Nyquist plot.
